# Development of a fast and user-friendly cryopreservation protocol for sweet potato genetic resources

**DOI:** 10.1038/s41598-020-70869-3

**Published:** 2020-09-07

**Authors:** Hannes Wilms, Natalia Fanega Sleziak, Maarten Van der Auweraer, Martijn Brands, Matthijs Verleije, Dirk Hardeman, Edwige Andre, Bart Panis

**Affiliations:** 1 Dept. Biosystems, Laboratory of Tropical Crop Improvement, KU Leuven, 3001 Leuven, Belgium; 2 Bioversity International, Belgian Office at KU Leuven, 3001 Leuven, Belgium

**Keywords:** Plant physiology, Plant sciences, Biodiversity

## Abstract

Sweet potato (*Ipomoea batatas*) is one of the ten most important staple crops and provides a livelihood for many people around the globe. To adapt to ever-changing circumstances farmers and breeders need to have access to a broad diversity of germplasm. This study focuses on the development of a cryopreservation protocol that allows the long term storage of different sweet potato cultivars. For this, a droplet vitrification protocol was optimized, comparing several parameters; preculture method (0.3 M sucrose vs no preculture); meristem position (axillary vs apical); plant age (3 to 9 weeks); regeneration medium (MS + 2.22 µM BA, Hirai and MS); and length of loading solution treatment (20 to 360 min). Two months after cryopreservation, the regeneration rates of the meristems were compared, which resulted in significant differences for the preculture method, meristem position and loading solution. With these new insights an optimized droplet vitrification protocol was developed with the following parameters: use of 3–9 week old axillary meristems, no preculture phase, 20 min LS treatment, 30 min PVS2 treatment, exposure to liquid nitrogen by droplet vitrification, warming treatment in RS for 15 min, 1 day 0.3 M sucrose recuperation culture, 1 month MS + 2.22 µM BA followed by 1 month of MS cultures. This protocol was subsequently tested on 10 representative accessions resulting in a post cryopreservation regeneration rate of more than 40% for 70% of the tested cultivars, showing that this protocol could be implemented for a large portion of existing sweet potato collections.

## Introduction

Sweet potato (*Ipomoea batatas* (L.)) was domesticated multiple times in different places in Central and South America more than 9,000 years ago^1,2^ and is currently grown in every continent, excluding Antarctica, for its tuberizing roots which are rich in carbohydrates and carotenes^3^. While sweet potato was originally only grown in the tropics, its cultivation has now spread to more temperate regions, like central Europe, where it is grown as an annual crop^4^. Each year, around 112 million tons of its storage root are produced globally. This makes sweet potato, in terms of production figures, the 15th most important crop in 2017, and if only staple foods are considered, it becomes the 8th most important (after, maize, wheat, rice, potatoes, soybean, cassava and bananas). Following the current trend, its production is suspected to further rise considerably in the coming years^5^.

The cultivation of sweet potato is hampered by different factors, such as drought, pest and disease, which can all cause severe crop losses. The two most common viruses, the sweet potato feathery mottle virus and the sweet potato chlorotic stunt virus, can cause the sweet potato virus disease when both are present in the plant causing a yield drop over 50%^6^. These viruses are often transmitted via vectors, e.g. white flies (*Bemisia tabaci* (Gennadius)), which enable them to spread easily from plant to plant^7^. Once infected, removal of the virus in the field is nearly impossible, eradication of the virus is only possible in in vitro plants and is practiced through, for example, cryotherapy, meristem culture and thermotherapy^8^ . Abiotic stress such as drought is suspected to become more severe due to climate change^9^.

The reaction to each of these stresses, however, is genotype-dependent. The different responses to drought stress can be explained by the broad range of adaptations that some cultivars have acquired over time, such as an adapted stomatal conductance or leaf area index^10^. Besides drought resistance, variation can also be observed in other traits like taste, colour and the response to other stresses such as virus infections—with some cultivars showing less or more severe symptoms^11^.

Access to this wide genetic diversity is important for farmers and breeders, as it allows them to adapt to changing environments. Resistant sweet potato cultivars could be introduced directly in regions that are plagued with certain diseases or indirectly by crossing them with other cultivars. National and international gene banks play an important role in this interaction between nature, farmers and breeders, since they provide easy access to such materials. The largest sweet potato genebank is hosted by the International Potato Center (CIP), with 8,054 accessions as reported in 2018^12^.

While seed conservation is considered as the most convenient method to store crops’ genetic resources, this is not always an option; there are plants that do not produce seeds (sterile crops like edible bananas), that produce non storable, recalcitrant seeds (cacao, avocado, coconut) or that, in the case of sweet potato, are clonally propagated—since its offspring does not have the same genetic set up when propagated by seed^13^. These clonally propagated crops have to be conserved in a different way and are therefore often kept in a field, greenhouse or in vitro collection^14^. The maintenance of such a collection, however, is labour intensive. Moreover, in vitro collections come with a cost and with the possibility that somaclonal variation will occur over time^15^.

Cryopreservation can solve these issues as it allows to store plant genetic resources at ultra-low temperatures where biological, enzymatic and chemical activities are halted. This causes the tissue to be suspended in time as long as it is kept at these temperatures^16^. For sweet potato, some reports have already been published on the development of cryopreservation protocols, using techniques such as vitrification^17^, encapsulation vitrification^18^, encapsulation dehydration^19^ and droplet vitrification^20–22^. These techniques all revolve around the prevention of ice crystal formation during cryopreservation, in the vitrification based techniques crystallization is prevented by vitrifying the explant by replacing the water that is present with cryoprotecting substances combined with rapid freezing, causing the water in the plant to go into an amorphous glass phase. Dehydration, meanwhile lessens the chance of freezing by removing as much water as possible without the addition of other molecules. In encapsulation based protocols the explant is encapsulated in an alginate bead before being cryopreserved. However, improvement is still needed, since many of the earlier reports only evaluated the survival and not the regeneration of the tissues^20^, or are using only a small selection of cultivars^18^. Vollmer and co-workers reported successful cryopreservation of apical meristems with a regeneration rate higher than 40%, in 38% of their 24 tested cultivars^21^.

The objective of this study was to develop a user-friendly cryopreservation protocol thereby improving regeneration rates of sweet potato plantlets after cryopreservation via droplet vitrification^23^. By optimizing different parameters (such as preculture, regeneration medium and meristem type) of the cryopreservation protocol and apply the optimized method to a range of cultivars that is representative of sweet potato diversity.

## Results

### Comparison of different propagation media

**Figure 1:**
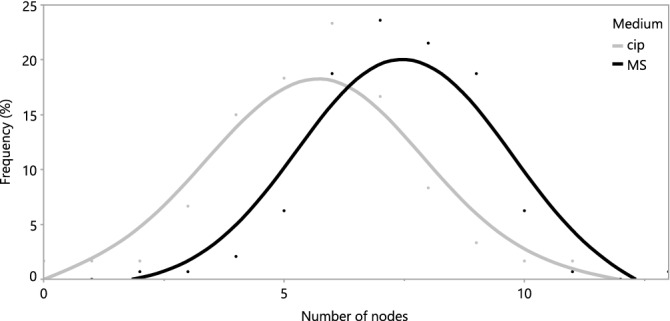
Frequency of the number of nodes of sweet potato plantlets cultured for 6 weeks on two different media; MS and CIP. The points represent the relative frequency of each observation and are an average of 4 cultivars (TIS, IBA, JEW and TAN). The curve was made by auto fitting a smooth curve in JMP.

The plantlets which were propagated on the two different media, Murashige and Skoog medium (MS) and CIP medium, differed significantly (P < 0.05) with respect to the number of new nodes that they produced after 6 weeks. This was both the case for all four cultivars combined and individually. The number of nodes on the plantlets grown on the MS tube medium resulted in one extra node per subculture compared to those grown on the CIP medium. These results are visualized in Fig. 1, where the range of both groups is approximated by a Gaussian distribution, with the curve peaking at 7 and 6 nodes for MS and CIP respectively. This higher number of nodes was also positively correlated with the average length of the plantlet, giving the plantlets grown on the MS medium longer shoots. There was also more callus observed on the plantlets on the CIP medium, but for all other visual markers (roots, leaf size, etc.), no significant differences were observed.

### Toxicity of loading solution (LS)

**Figure 2:**
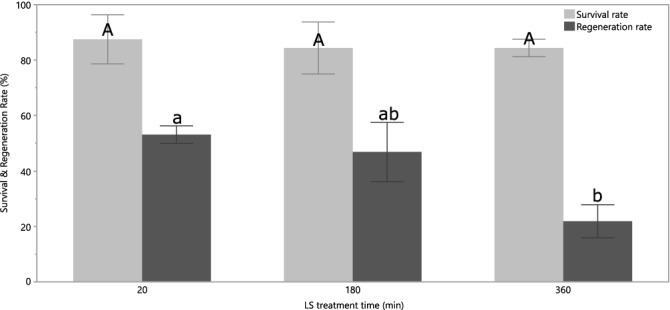
Survival and regeneration rates of sweet potato meristems from 4 cultivars (TIS, IBA, JEW and TAN) after different LS treatment times. The error bars represent the standard error.

The three different LS durations did not significantly influence the survival rates of the meristems (Fig. 2). Nevertheless, when compared to the control plants, that were not treated with LS, a ~ 10% decrease was observed (results not shown). The regeneration rate (~ 50%) of the meristems that were treated with LS for 180 min did not differ significantly with those that were only treated 20 min. However, when this was increased to 360 min, a significant decrease could be observed (~ 20%) (Fig. 2). While the 20 and 180 min treatment did not differ significantly in respect to their final regeneration rates, the 180 min treatment resulted in a slower growth of the meristems.

### The effect of plant age

A significant difference between the survival rates of the non-cryopreserved meristems that were excised from 3- week-old and 6-week-old plantlets was observed, with later plantlets reacting better to the treatment (Table 1).However, such a plant age effect could not be statistically proven for all other parameters. This shows that apical and axillary nodes of different ages, within the tested timeframe, can be used for cryopreservation.

**Table 1:** Comparison of survival and regeneration rates 8 weeks after cryopreservation of axillary and apical meristems from 4 different cultivars (JEW, IBA, MAN and CMR) that were excised from plantlets of 3 different ages.

Plantlet age	Survival % (Mean ± standard error)		Regeneration % (Mean ± standard error)	
	Control	Cryopreserved	Control	Cryopreserved
3 weeks	81.8 ± 4.0 a	87.6 ± 3.9 a	21.3 ± 5.2 a	29.6 ± 4.8 a
6 weeks	91.5 ± 2.7 b	92.7 ± 1.1 a	28.1 ± 4.9 a	39.7 ± 5.8 a
9 weeks	85.1 ± 2.5 ab	88.0 ± 2.4 a	23.0 ± 6.2 a	34.8 ± 4.6 a

Significant differences in a column are shown with a different letter. Controls and Cryopreserved are viewed as 2 different groups.

### The effect of a 0.3 M sucrose preculture

**Figure 3:**
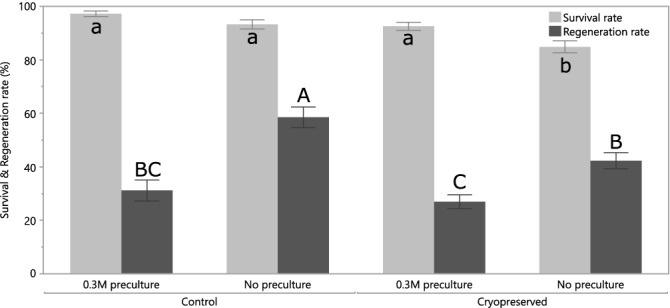
Effect of a 0.3 M sucrose preculture of axillary and apical meristems of 3 different cultivars (IBA, CIN and CMR) on the survival and regeneration rates after cryopreservation. Significant differences in the survival (lowercase) or regeneration (upper case) rates are annotated with a different letter. The error bars represent standard error.

A 1-day preculture on 0.3 M sucrose shows a significant positive effect on the survival rate of both control and cryopreserved meristems (Fig. 3). However, for the regeneration rate the opposite is observed. This could be due to the fact that the preculture also increases the cryopreservation ability of more hydrated non-meristematic (and thus non-totipotent) cells. It could thus become possible that a surviving leaf primordia can regenerate into a leaf without the presence of a surviving meristem. This might explain the discrepancy between the survival and regeneration rate.

### The effect of meristem type

**Figure 4:**
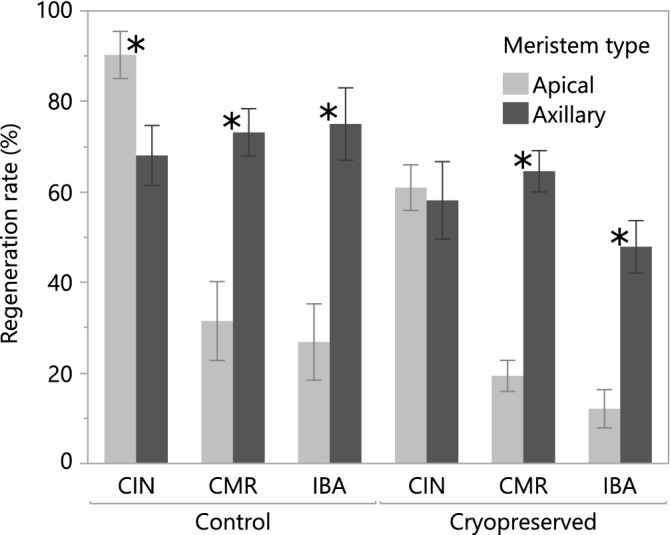
Comparison between the regeneration rate of axillary (ax) and apical (ap) sweet potato meristems of 3 cultivars; CIN, CMR and IBA. Significant differences between the regeneration rates of apical and axillary meristems are indicated with a “*”. The error bars represent standard error.

The overall survival rate showed a significant difference between the two groups, with axillary meristems showing an overall higher rate (~ 7% more, results not shown). On a cultivar level, some differences among the 3 cultivars could be observed. The overall regeneration rate of the axillary meristems showed a similarly significant behaviour and were significantly better than that of the apical meristems on both cryopreserved and control samples. On cultivar level there was a similar trend to the survival rate where CMR and IBA show the same significant improvement when using axillary meristems compared to apical ones, while this is not the case for CIN (Fig. 4). These results show that a cryopreservation protocol for sweet potato preferably uses axillary meristems because (i) post-thaw regeneration rates are generally higher and (ii) more meristems can be excised from one plant (6 axillary meristems from a six-week old plant and only one apical).

### The effect of regeneration medium

Both control and Cryopreserved meristems survive significantly better on the 2.22 µM BA compared to the other media, with a survival rate of 96.8 and 69.2% respectively. This effect was observed on 3 of the 4 cultivars tested (results not shown), only in case of IBA no significant differences were observed. The regeneration rates, however, did not show any significant differences resulting in an average regeneration rate over all media of 29% and 82% for the cryopreserved and controls respectively (Table 2).

**Table 2:** Comparison of the survival and regeneration rate of 4 cultivars (TAN, JEW, IBA and TIS) 8 weeks after cryopreservation of 3 different media.

Treatment	Survival% (Mean ± standard deviation)		Regeneration% (Mean ± standard deviation)	
	Control	Cryopreserved	Control	Cryopreserved
2.22 µM Ba	96.8 ± 9.9 a	69.2 ± 32.4 a	83.1 ± 24.6 a	26.0 ± 29.1 a
Hirai*	86.9 ± 20.8 b	46.2 ± 38.9 b	84.1 ± 23.3 a	33.4 ± 35.3 a
MS	78.1 ± 25.7 c	33.3 ± 34.0 b	77.7 ± 26.2 a	28.4 ± 33.9 a

Significantly different numbers in each column are annotated by different letters.

**Figure 5:**
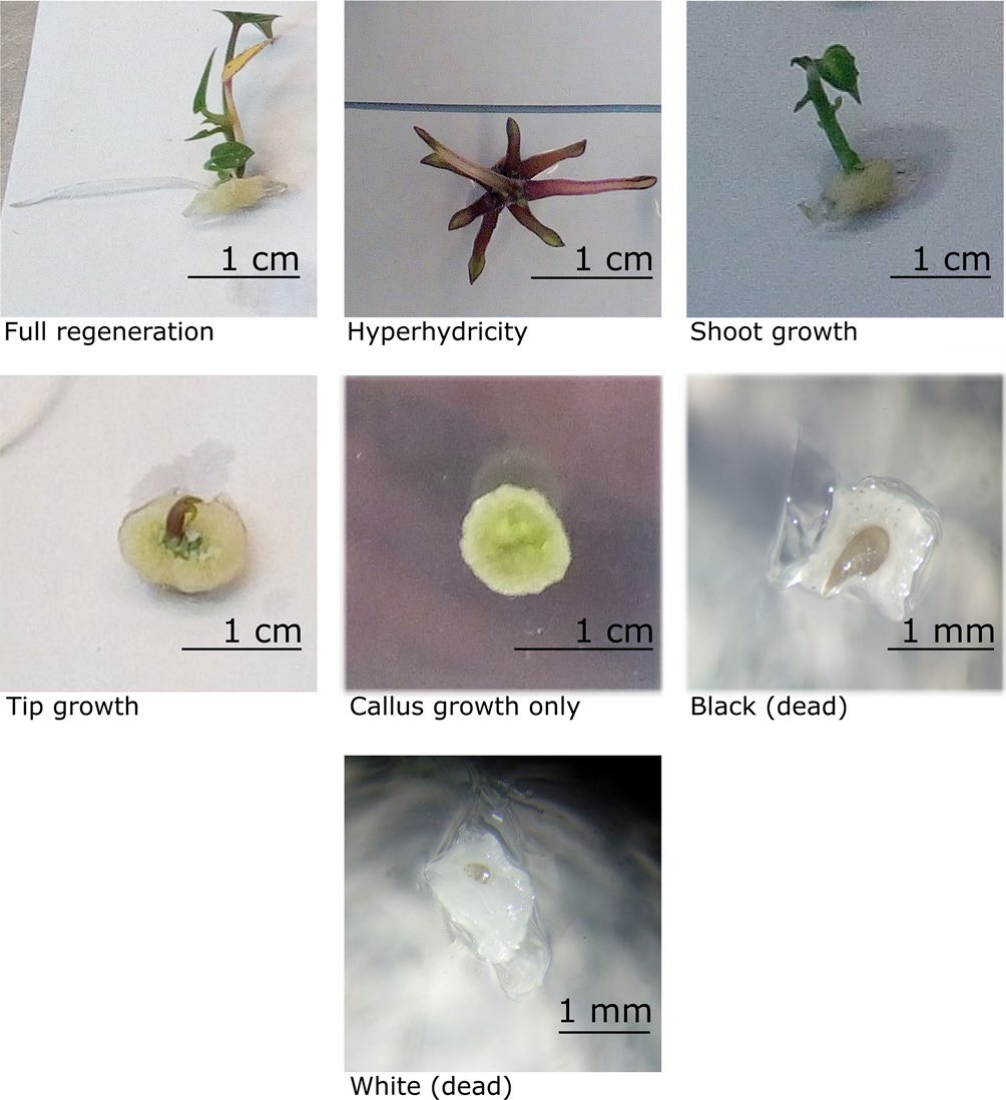
Seven categories of post thaw reactions. 1: Full regeneration, 2: Hyperhydricity, 3: Shoot growth, 4: Tip growth, 5: Callus growth only, 6: Black (dead), 7: White (dead).

A general observation, however, was that meristems growing on 2.22 µM BA produced a lot of callus. This callus, however, originated from the wound tissue beneath the meristem and not from the meristem itself (examples are shown in Fig. 5). The shoots that thus emerge do not originate from callus tissue but from the original meristem, an observation that is important for conservation purposes, as shoots originating from callus tissue are more prone to somaclonal variation.

### Application of the optimised protocol to ten different cultivars

**Figure 6:**
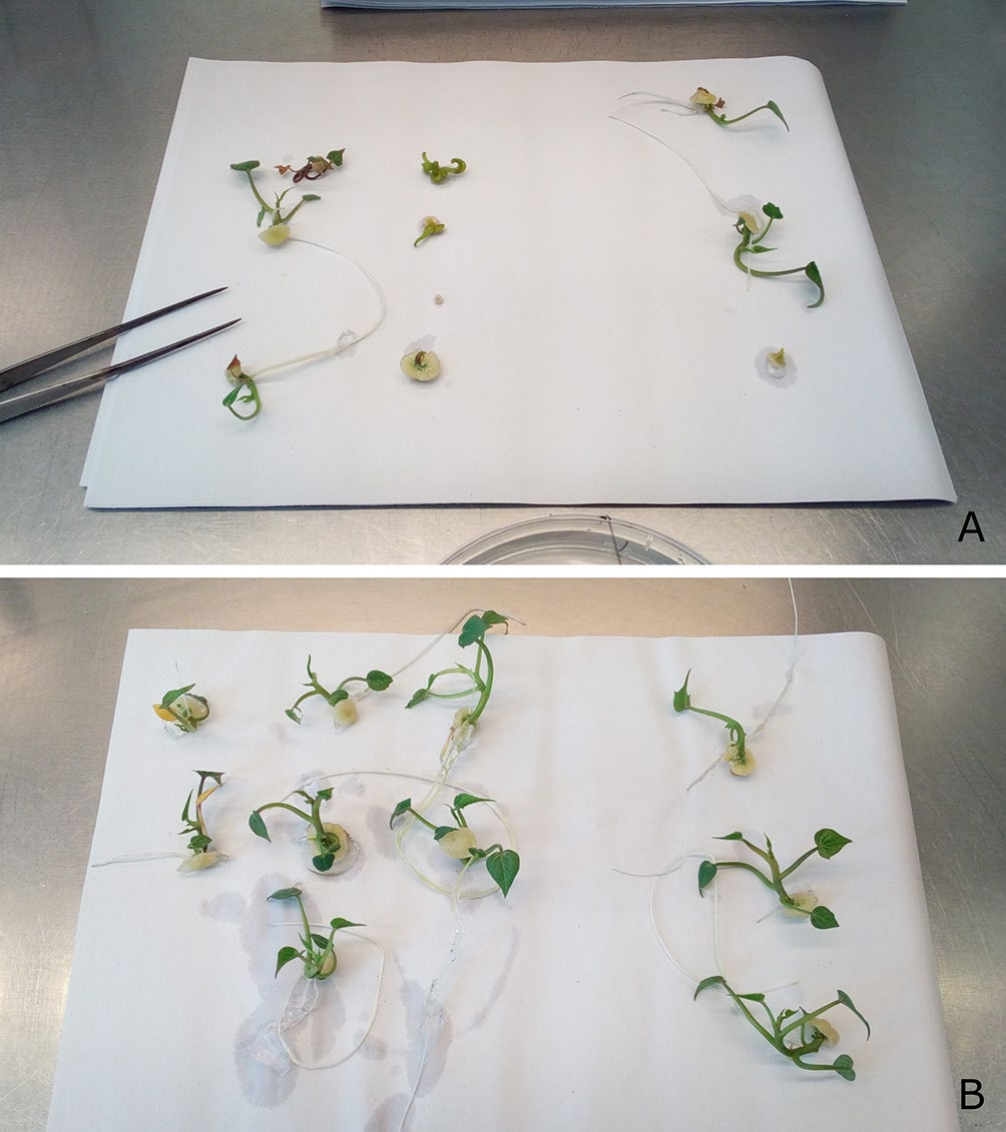
Plantlets 2 months after cryopreservation presented on a A5 paper. (**A**) 7 cryopreserved meristems with ranging regeneration results (left) and 3 control plantlets (right) from the CMR cultivar. (**B**) 7 cryopreserved and fully regenerated meristems (left) next to 3 control plantlets (right) from the TAN cultivar.

Based on the results of the experiments described above, an optimal droplet cryopreservation method is formulated using the following parameters: use of axillary meristems; no preculture; LS treatment 20 min; PVS2 treatments 30 min; RS treatment 15 min and the use of a 2.22 µM BA regeneration medium. This protocol was subsequently tested on a broad range of cultivars. Recovery for 2 cultivars, CMR and TAN is shown in Fig. 6.

Different regeneration rates for the different cultivars were observed (Table 3). Three different groups according to their regeneration rate can be distinguished. The first group, consisting of CMR, IBA, TAN and TRUJ, responds satisfactorily to the developed cryopreservation protocol and could be considered as easily cryopreservable, with post-cryopreservation regeneration percentages ranging between 58 and 85%. The second group consists of CIN, ESP and MAN, which are intermediately cryopreservable and show regeneration rates around 50%, a rate that is considered high enough for cryobanking. The third group consists of the difficult to cryopreserve cultivars (CAM, JEW and TIS) with low regeneration levels ranging between 9 and 22%. .

**Table 3:** Survival and regeneration rate of 10 sweet potato cultivars 8 weeks after cryopreservation; significant differences within a column are annotated with a different letter.

Cultivar	Survival% Mean ± standard deviation		Regeneration% Mean ± standard deviation	
	Control	Cryopreserved	Control	Cryopreserved
CAM	81.5 ± 24.2 a	54.2 ± 23.0 abcd	29.6 ± 35.1 b	22.2 ± 14.5 bc
CIN	94.4 ± 13.0 a	89.4 ± 14.8 ab	73.6 ± 21.9 a	56.1 ± 30.4 ab
CMR	97.8 ± 8.6 a	91.1 ± 10.5 a	76.7 ± 21.6 a	62.0 ± 17.0 a
ESP	92.6 ± 14.7 a	54.0 ± 31.8 bcd	74.1 ± 22.2 a	47.6 ± 27.7 ab
IBA	97.8 ± 8.6 a	84.1 ± 19.6 ab	71.1 ± 29.9 a	58.5 ± 25.7 a
JEW	55.6 ± 47.1 b	39.7 ± 40.3 cd	22.2 ± 37.3 b	9.5 ± 16.0 c
MAN	90.7 ± 18.8 a	59.0 ± 19.9 abc	72.2 ± 33.3 a	49.5 ± 22.1 ab
TAN	100.0 ± 0.0 a	87.3 ± 15.1 ab	100.0 ± 0.0 a	83.9 ± 18.1 a
TIS	92.6 ± 14.7 a	30.2 ± 31.5 d	70.4 ± 35.1 a	11.1 ± 17.2 c
TRUJ	100.0 ± 0.0 a	90.5 ± 14.3 ab	85.2 ± 24.2 a	81.0 ± 17.5 a

## Discussion

Droplet vitrification for the cryopreservation of organized plant tissues can be considered as a generic cryopreservation method as it has already been applied to many different unrelated crops, such as banana^24^ , magnolia^25^, Arabian pea^26^ , garlic^27^ and apple^28^. Its main advantage, compared to other methods, such as encapsulation and slow rate cooling, lies in its simplicity and time efficiency^29^. Nevertheless, this method always needs to be adjusted to the different plant species and tissues. Panis and co-workers developed a methodology to determine optimal parameters for each species, following a step-by-step plan^23^. These parameters include: optimize the quality of the starting material including absence of endophytes, define a suitable multiplication medium, define a meristem tip regeneration medium, screen LS toxicity and PVS2 toxicity at 0 °C. This research focuses on some of these parameters, since earlier reports already determined a few of these parameters, e.g. the optimal PVS2 length (30 min)^21,30^.

The steady supply of material of high quality is a prerequisite to a cryopreservation protocol with a high reliability In this research, material that was already sterile and devoid of endophytes, was acquired from CIP, which was then further propagated. Since the amount of material increases more than six fold every 6 weeks with the propagation method, there is no need for another mass propagation method such as (in)direct organogenesis or embryogenesis, as these tend to show a higher risk at somaclonal variation^31,32^. The MS medium that we applied increased plant growth compared to the CIP medium. These results are contra- intuitive since the addition of gibberellic acid to a culture medium results for most of the plant species in an elongation of the internodes and therefore results in longer plants^33^. However this growth discrepancy could be explained by the lower nutrient concentration that is present in the CIP medium (MS vs half strength MS), which is in line with the results reported by Arrigoni-Blank and co-workers^34^.

For our tested time window of 3 till 9 weeks after subculture, no significant differences were found that are related to the age of the material in present study. This means that for genebanking purposes, more material is readily available since plantlets of different ages can be used. The utilization of older material also provides more meristems per plantlet; From a 6 week old plantlet on average 7 meristems can be excised, which is significantly more than a from 3 weeks old plant with, on average 5 meristems. Nonetheless, the age can still be an important parameter for other crops. This is especially true in woody plants where older meristems change their cell wall composition over time, which can either result in an increase of regeneration in the case of apple buds^28^ or a decrease in the case of silver birch^35^. This age effect is also present in several non-woody plants such as Chrysanthemum^36^ and potato^37^.

The meristem type did show to significantly affect the regeneration rate after cryopreservation, with the use of axillary meristems resulting in higher regeneration rates than their apical counterpart. However, the magnitude of this effect was shown to be cultivar dependent. Other plant species show varying results; axillary meristems are optimal for Chrysanthemum^36^ while in the case of Dianthus, apical meristems give better results^38^. While we can see that this clearly leads to different outcomes, reports comparing these two kinds of meristems (including sweet potato) are rare. Another advantage in using axillary meristems is the number of meristems per plant that can be used, depending on the age, 6 or more meristems per plant are available.

In many plant species and multiple plant cryopreservation protocols, a preculture phase is applied to increase the regeneration rate of the cryopreserved meristems. In this study, a 0.3 M sucrose preculture shows a significant improvement on the survival rate, which is not mirrored by an increase in the regeneration. The higher survival rate could be caused by the increased cryopreservability of more water containing non-meristematic cells that normally do not survive without a sucrose preculture. These cells form callus and overgrow the surviving meristem thereby “suffocating” it. This could explain the drop in regeneration; however, this decrease is in conflict with earlier reports in sweet potato^22,39^.

No significant differences between the regeneration rates of the plantlets on the three different media were observed; however, this was again not the case for the survival rate. This difference could be due to the BA in the medium which helps non-meristematic cells grow and form callus and which might explain the higher survival rate. In other reports on sweet potato cryopreservation, BA is used multiple times. However, it is often combined with other growth regulators^17^, or applied for a shorter amount of time (~ 7 days)^18^. Such adaptations could reduce the growth of callus, yet we determined that the callus growth did not interfere with the growth of the meristem into a full grown plantlet. The callus growth induced by BA, is thus not considered as a negative factor, so further optimization of this step is not a priority.

While LS is important to increase resistance toward the toxic effects of the vitrification solutions, it also induces toxicity after prolonged exposure. These observations are in line with reports on other crops where a decrease in regeneration has also been observed. The severity of this effect is very crop dependent; *Citrus madurensis* already shows a steep decrease in regeneration after 30 minutes^40^ while for banana the LS treatment time can be stretched for at least 7 hours^24^. Other reports of sweet potato suggest a longer treatment time (1 h) as an ideal instead of the 20 min^18^. However these times cannot really be compared, since the latter involves encapsulation and this generally requires longer treatment times than droplet vitrification due to the need for the solutions to diffuse into the beads. Provided the toxicity would have been insignificant for longer periods, our cryopreservation protocol could have been simplified and excised meristems could have been directly stored in the LS after excision. However since prolonged exposure induces a toxic effect this is not an option.

We observed that sweet potato cultivars react very differently to the cryopreservation protocol. Some of the cultivars achieved a regeneration rate of more than 80% while others barely reached 10%. One would assume that a cryopreservation method would have a similar effect on plants of the same species, however our results are in agreement with literature. Multiple plant species, including sweet potato, already showed huge variation within their species, regarding cryopreservation ability. Nevertheless, it would be interesting to link drought resistance data of these sweet potato plants in the field with their cryopreservation ability. It was proven before, that post-thaw regeneration rates of banana, apple and cassava in the field could be correlated with variety characteristics^24,41,42^. While many drought resistance experiments have been executed on a wide variety of sweet potato cultivars; results including one of our 10 studied cultivars are scarce. Omotobora et al. included 2 of the cultivars, JEW and TAN, in a series of preliminary drought experiments that were executed on 50 accessions. JEW and TAN proved in their pre-screening trials to be intermediate tolerant and susceptible to droughts respectively^43^, which contrasts with the earlier notion that more drought resistant plants cryopreserve better in general.

While seven out of ten cultivars reacted satisfactory on the optimized cryopreservation treatment, 3 of them still show a regeneration rate that is critical for cryobanking. All three show a similar low regeneration rate after cryopreservation but the reason of this varies greatly, suggesting that developing one method for all accessions might not be feasible. For instance, CAM meristems show a lot of problems with hyperhydricity, likely due to the long BA treatment^44^. This could be overcome by shortening the BA treatment or decrease the BA concentration in the medium. TIS meristems on the other hand, show good growth of control meristems but this is not reflected in the cryopreserved meristems in contradiction with the other cryopreservable cultivars. The main bottleneck here was thus, the cryopreservation step itself. For this cultivar an alternative vitrification solution or treatment time could be applied, as the meristem might not be protected (dehydrated) enough. Finally JEW also shows a low regeneration rate in its non-cryopreserved control, suggesting that the problems might be more tissue culture related in general or it could be related to toxicity towards the PVS2 treatment. Observation during the regular subculture also showed that this cultivar was growing much slower compared to the other cultivars, adding credit to this hypothesis. This means that further improvement is still possible.

The regeneration rate of the optimized droplet vitrification protocol reached more than 40% for 7 out of 10 tested cultivars. These regeneration rates combined with the simplicity of the protocol (no need of a preculture, or extra encapsulation step, the fact that by using axillary meristems more material is available, etc.), make it an acceptable and reliable protocol for genebanking purposes of sweet potato. A trained technician following this protocol can excise and cryopreserve more than 180 meristems in one day. These results improve on earlier reports in several ways. Firstly, the use of meristematic tissue limits the chance of somaclonal variation which is not desirable for conservation purposes. The droplet vitrification of meristems thus contrasts with early cryopreservation protocols which made use of embryogenic tissues^19^ . Secondly, the cryopreservation protocol was tested on more cultivars than most previous studies. Some previous studies only used one^20,22^, two^17^ or three^18,45^ cultivars, which might result in biases when interpreting the results and overgeneralizations, since this report shows that the cultivar type is a very important parameter, with 83.9 and 9.5% regeneration rate of the best and worst cultivar respectively. However, there is a trend in using multiple cultivars (≥ 5) when reporting on cryopreservation protocols^21,46^ . Thirdly, the research makes use of a well-defined post-cryopreservation reaction categorization, which is a valuable addition to the survival rate or “shoot elongation” often used in earlier reports^20,46,47^. This distinction is important since we show that survival does not equal plantlet regeneration, sometimes it is not even correlated. There are different stages where shoot growth stops and does not result in fully regenerated plantlets. This problem has also been observed in other crops, e.g. cassava^48^ and potato^49^. To confirm plant regeneration after cryopreservation, regeneration should be followed over a longer period, to avoid false positives. Fourthly, it is an easily executable protocol with less steps, as it requires no preculture (≥ 16 h)^18^ or encapsulation (~ 30 min)^18^, and the cryoprotective steps (LS & PVS; total of 50 min in present method) take less time compared to those of encapsulation dehydration (~ 4 h)^18^ and plate vitrification (~ 2 h), making it possible to treat more meristems per day. Lastly, we report higher regeneration rates compared to similar studies on sweet potato, taking into account the parameters; use of meristematic tissue, use of multiple cultivars, correct use of the term regeneration and, use of droplet vitrification. Vollmer and co-workers followed a similar protocol on 24 different accessions, but with some key differences such as a different preculture and apical meristems, which have been shown here to significantly influence the regeneration, this resulted in regeneration rates between 1.7 and 68.5%^21^. However, to truly compare with our results, the same cultivars should be tested.

## Conclusion

We can conclude that firstly the use of axillary meristems for the cryopreservation of sweet potato is an interesting option and could even be an improvement for other plant species. Secondly, the optimized droplet vitrification protocol showed regeneration rates of cryopreserved meristems ranging from 9.5 to 83.9%, with 70% of tested cultivars having regeneration rates higher than 45%. While this protocol should still be optimized for certain “difficult” accessions, we conclude that this protocol is applicable for long-term conservation for most of the sweet potato collections.

## Materials and methods

### Plant material

In vitro-grown plantlets of ten different *Ipomoea batatas* cultivars; Camote Mata Serrano (CAM; Cip-420530); Cinitavo (CIN,Cip-440669); CMR 1112 (CMR; Cip-440145); Espelma (ESP, Cip-421028); Ibarreno (IBA; Cip-400989); Jewel (JEW; Cip-440031); Manchester Hawk (MAN; Cip-400040); Tanzania (TAN; Cip-440166); Tis 87/0029 (TIS; Cip-442764); and Trujillano (TRUJ; Cip-420665) were supplied by CIP (Lima, Peru). These ten cultivars were chosen to represent the diversity of the cultivars present at the sweet potato genebank of CIP as they originate from a broad range of countries over 4 different continents.

### Plant multiplication

In vitro plantlets were propagated on “CIP medium” and plain MS medium^50^). The CIP medium contains half strength MS salts (Duchefa Biochemie, M0221) supplemented with 30 g/L sucrose, 2.8 g/L gelrite, 2 mg/L calciumpanthotenate, 100 mg/L calciumnitrate, 200 mg/L ascorbic acid and 10 mg/L gibberellic acid, pH was set to 6.12 before autoclavation (b.a.). The MS medium contains MS salts and vitamins (Duchefa Biochemie, M0222) supplemented with 25 g/L sucrose and 2.8 g/L gelrite, pH was set to 6.12 b.a.. Nodal fragments from the in vitro plantlets were excised in a sterile laminar flow bench. This was done by removing the leaves and roots of the plantlet, after which 1 cm stem fragments were cut, each containing one axillary meristem in the middle. Three fragments were transferred to each culture tube and grown in a 24 °C growth room on a 16/8 h light/dark regime with the light being provided with 36 W (cool white)/ 840 Lumilux fluorescent lights. The material was subcultured every 5–6 weeks.

Six weeks after initiation, the number of new nodes was counted. This experiment was initially executed on the following 4 cultivars: TIS, IBA, JEW and TAN. Further propagation of all 10 cultivars was done with the medium that proved to be the most productive.

### Meristem excision

**Figure 7:**
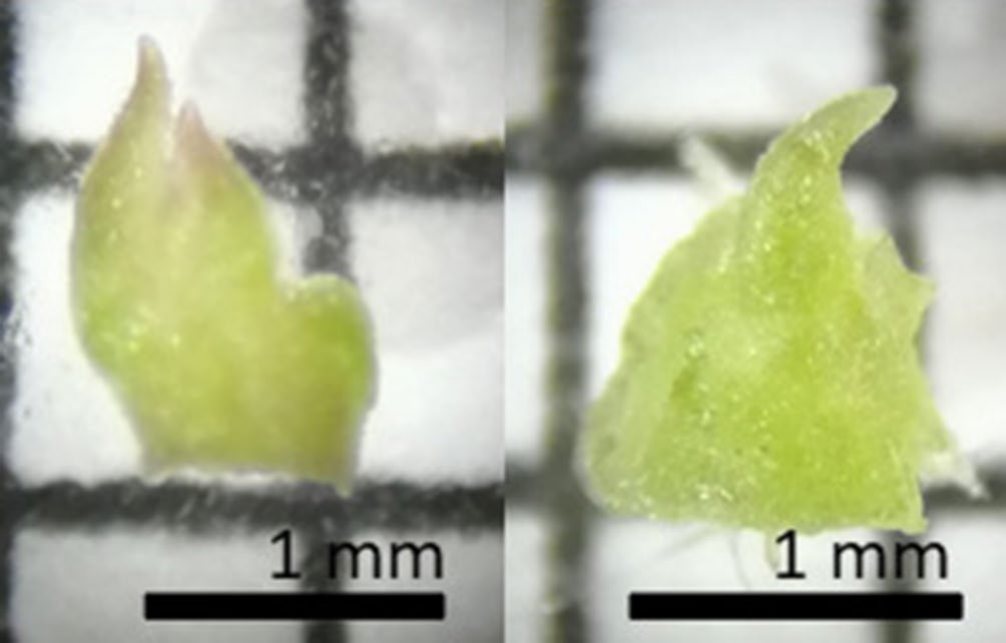
Excised apical (left) and axillary meristem (right) from a CIN and ESP cultivar plantlet respectively, displayed on millimetre paper. The black bar represents 1 mm.

Two meristem types were excised using a binocular microscope; apical and axillary meristems. The apical meristems were excised by trimming the top leaves until the apical meristem is visible. Then a cut is made in the stem underneath the apical meristem, leaving the apical dome with 2 to 4 leaf primordia (Fig. 7). The axillary meristems were excised by first removing the leaves completely, including the petiole. From this a small cube of 1mm^3^ was excised containing the axillary meristem on one of the sides (Fig. 7) A movie demonstrating this process is added as supplementary information in the digital version of this paper (see Online Supplementary Resource 1). The exact position of the axillary meristem on the stem was not taken into account in this research, since it was proven that this had no significant impact on the survival rate after cryopreservation^39^.

### Preculture

In the “no preculture method”, the excised meristems are transferred on top of a sterile filter paper placed on a MS plate (MS, 30 g/L sucrose and 3 g/L gelrite, pH set to 6.12 b.a.). As soon as sufficient meristems for that specific experiment are excised, they are subjected on the same day to the cryopreservation procedure. In the “preculture method”, the excised meristems are transferred on top of a sterile filter paper placed on a 0.3 M MS plate (MS, 102.7 g/L sucrose; 3 g/L gelrite, pH set to 6.12 b.a.) and kept on this medium in the dark for14 to 16 h before cryopreservation takes place.

The difference between precultured and non-precultured meristems was tested by comparing the post-thaw survival and regeneration rate of 950 of both axillary and apical meristems originating from 3 different cultivars (IBA, CIN, and CMR). These were cryopreserved via the droplet cryopreservation protocol using the following parameters: Loading solution (LS) 20 min; Plant Vitrification Solution (PVS2) 30 min; Recovery Solution (RS) 15 min and 2.22 µM BA regeneration medium.

### Droplet cryopreservation

Precultured or non-precultured meristems were transferred to a sterile 30 ml plastic tube containing 15 ml of LS ( MS supplemented with 2 M glycerol and 0.4 M sucrose; pH 5.8), where they remain at room temperature for 20 min. Then the LS was removed from the tube with a sterile plastic boll pipette, taking care not to remove or damage the meristems.

The empty tube was then filled with 15 ml chilled PVS2^51^ with the Murashige- Tucker medium replaced by MS ( MS supplemented with 30% glycerol, 15% ethylene glycol, 15% DMSO and 0.4 M sucrose, pH 5.8) and subsequently placed in an ice bath for 30 min^21,23^.

Of each sample of 10 meristems, 3 were directly transferred from the PVS2 to the RS (MS supplemented with 1.2 M sucrose, pH 5.8) at room temperature to act as a control. The remaining 7 meristems were transferred with a plastic boll pipet to a sterile aluminium foil strip (4 × 15 mm). From this strip, the remaining PVS2 fluid was removed until only a thin layer of PVS2 surrounds each meristem. Subsequently the aluminium strip was plunged into liquid nitrogen (LN). When the LN surrounding the aluminium strip stopped boiling, the strip containing the meristems was transferred to a 2 mL cryotube filled with liquid nitrogen where the meristems remained for at least 30 min. To warm the meristems, the aluminium strip with meristems was removed from the cryotube with liquid nitrogen and directly plunged in the RS at room temperature.

Both control and cryopreserved meristems were exposed to the RS for 15 min. Following this, they were placed one by one with a plastic boll pipette onto a filter paper placed on a MS plate containing 0.3 M sucrose. The plates were then sealed with parafilm and stored overnight in darkness at a temperature of 24 °C. The next day, the meristems were transferred on to the regeneration medium in an upright position, with the meristematic domes not fully submerged in the medium. The meristems were left in the dark for 7 days, where after they were moved into the light.

**Figure 8:**
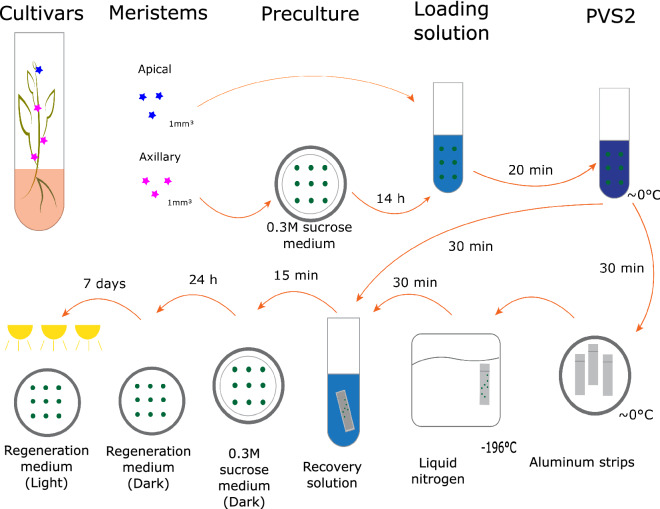
Step-by-step cryopreservation process of sweet potato.

After 1 month they were moved from the regeneration medium to new MS plates (Fig. 8).

### Effect of the age of the in vitro plantlet

The effect of plant age was tested by comparing the post-thaw regeneration rates of 215 meristems, for both apical and axillary each, from 3, 6 and 9 weeks old plantlets from the JEW, IBA, MAN and CMR cultivars, using the following parameters: preculture (0.3 M sucrose); 20 min treatment with LS; 30 min with PVS2; 15 min with RS and regeneration on 0.3 M sucrose medium (1 day) followed by MS with 2.22 µM 6-Benzyladenine (BA) regeneration medium.

### Effect of toxicity of the loading solution

For this experiment 24 apical and 72 axillary meristems of the cultivars TIS, TAN, IBA and JEW were subjected to 3 different LS treatment times (20, 180 and 360 min). After the LS treatment the meristems were transferred to a MS plate containing 0.3 M sucrose for one day plate after which they were transferred to an MS plate. Thereafter, the survival and regeneration rates were compared.

### Effect of composition of the regeneration media

Three different media were tested: MS (MS supplemented with 25 g/L sucrose and 2.8 g/L gelrite), Hirai* (MS supplemented with 30 g/L sucrose, 1 g/L casein hydrolysate, gibberellic acid 0.5 mg/L and 2 g/L gelrite; which is a slightly altered medium of Hirai and Sakai^18^, and 2.22 µM BA (MS supplemented with 25 g/L sucrose; 2.8 g/L gelrite and 2.22 µM BA). The pH of the media were set to 6.12 b.a. These media were already previously reported to have been used successfully for cryopreservation of sweet potato meristems. The survival and regeneration rate of these media after cryopreservation were compared for 939 apical and 939 axillary meristems from 4 cultivars (TIS, JEW, IBA and TAN). The cryopreservation parameters were: No preculture; LS 20 min; PVS2 15 min; RS 15 min.

### Effect of axillary versus apical meristem

This was tested by comparing the post-thaw regeneration rate of 475 apical and 475 axillary meristems originating from 3 different cultivars (IBA, CIN, and CMR). These were cryopreserved using the following parameters: No preculture; LS 20 min; PVS2 30 min; RS 15 min and 2.22 µM BA regeneration medium.

### Post-cryopreservation regrowth

Observations were executed one and two months after cryopreservation using a binocular microscope.

To express the results of the regrowth(survival) and regeneration, 7 categories of post thaw reactions are distinguished. In case of doubt, the lower growth category is taken in order to avoid false positives. The categories are summarized below and a visual representation of a typical meristem in each of the 7 categories is shown in Fig. 5.

*Full regeneration* (F) are those meristems that have grown multiple leaves, each containing a new meristem in the axil, and that are growing visible roots. These plantlets are able to regenerate into a new plant that can be subcultured and transferred to the soil. A *Hyperhydricity* (H) score is given to meristems which do form new leaves and meristems, but are growing abnormally. These plantlets have narrow leaves with a thick stem and roots that grow upwards. These are not categorized as regeneration as subculturing these plants will not lead to plantlets that can be transferred to the field. *Shoot growth* (S) is linked to meristems that produced a limited number of leaves, around 3, and then stop growing. They remain rootless. *Tip growth* (T) means that the meristem is visibly growing but shows no unfolded leafs. A *callus* (C) score is given when there is no visible growth other than callus. *Black* (B) or *White* (W) is given to meristems that have died either after or before/during cryopreservation. In many cases, callus growth is associated with one of the above categories.

To calculate the post thaw regeneration rate of a plate, the Full regeneration (F) meristems were counted and divided by the total number of meristems on the plate. The survival rate was calculated by counting all meristems with living tissue (F, H, S, T and C).

### Statistical analysis

The comparison of the number of nodes after 6 weeks on the 2 propagation media was executed using a one-sided tail, student t-test (homoscedastic) with a P-value < 0.05, when the variances were considered equal. When the variances were not considered equal, the student t-test (heteroscedastic) was performed.

Excel was used to collect the data from the various experiments. The raw data that was obtained in these experiments, has been made available on the Mendeley data repository^52^. The regeneration and survival data were transformed using the formula $y = \arcsin \left( {\sqrt {\frac{x}{{100}}} } \right)$ to create a normal distribution, wherein x = regeneration rate (%) or survival rate (%). This data was then analysed using the JMP statistical software to identify significant factors on a P < 0.05 level with an ANOVA test in combination with a Tukey HSD test.

## Supplementary information

Supplementary file1

## Data Availability

The datasets generated during and/or analysed during the current study will be available in the Mendeley data repository under the name: “Supplementary dataset for: Development of a fast and user-friendly cryopreservation protocol for sweet potato genetic resources”, at the doi link: https://dx.doi.org/10.17632/r4pcc3g4gn.1
